# High-resolution spatiotemporal pH_e_ and pO_2_ imaging in head and neck and oesophageal carcinoma cells

**DOI:** 10.1186/s40170-021-00257-6

**Published:** 2021-05-04

**Authors:** Alexandra Blancke Soares, Robert Meier, Gregor Liebsch, Sabina Schwenk-Zieger, Martin E. Kirmaier, Sebastian Theurich, Magdalena Widmann, Martin Canis, Olivier Gires, Frank Haubner

**Affiliations:** 1 Department of Otorhinolaryngology, Head and Neck Surgery, University Hospital, LMU Munich, Marchioninistr. 15, 81377 Munich, Germany; 2 PreSens Precision Sensing GmbH, Am Biopark 11, 93053 Regensburg, Germany; 3 Clinical Cooperation Group “Personalized Radiotherapy in Head and Neck Cancer”, Helmholtz Zentrum München, German Research Center for Environmental Health GmbH, 85764 Neuherberg, Germany; 4 Department of Medicine III, LMU University Hospital, Ludwig Maximilians University Munich, 81377 Munich, Germany; 5 Cancer and Immunometabolism Research Group, Gene Center LMU, Ludwig Maximilians University Munich, 81377 Munich, Germany

**Keywords:** OCR, ECAR, Spatiotemporal pO_2_ and pH quantification (STO_2_p-Q), Ratiometric fluorescence imaging, VisiSens technology, Head and neck squamous cell carcinoma, Oesophageal cell carcinomas, Cancer cell metabolism

## Abstract

**Background:**

pO_2_ and pH are physiological parameters relevant for different processes in health and disease, including wound healing and cancer progression. Head and neck squamous cell carcinomas (HNSCC) and oesophageal squamous cell carcinomas (ESCC) have a high rate of local recurrence that is partly related to treatment-resistant residual tumour cells. Hence, novel diagnostic tools are required to visualise potential residual tumour cells and thereby improve treatment outcome for HNSCC and ESCC patients. We developed a device to spatiotemporally measure oxygen consumption rates (OCR) and extracellular acidification rates (ECAR) to distinguish HNSCC and ESCC cells from healthy cells in vitro, exploiting general metabolic differences between cancer cells and healthy cells.

**Methods:**

OCR and ECAR were measured via a newly developed device named STO_2_p-Q (*S*patio*T*emporal *O*_*2*_ and *p*H *Q*uantification) using the VisiSens technology based on ratiometric fluorescence imaging, facilitating spatiotemporal resolution. Results were confirmed using extracellular flux analyses (Seahorse technology).

**Results:**

STO_2_p-Q is described and used to measure OCR and ECAR in HNSCC and ESCC cell lines and normal fibroblast and epithelial cells as components of the tumour microenvironment. OCR measurements showed differences amongst HNSCC and ESCC cell lines and between HNSCC/ESCC and normal cells, which on average had lower OCR than HNSCC/ESCC cells. Both OCR and ECAR measurements were independently verified using the Seahorse technology. Additionally, using STO_2_p-Q, HNSCC/ESCC, and normal cells could be spatially resolved with a resolution in the low millimetre range.

**Conclusions:**

We developed a method to spatiotemporally measure OCR and ECAR of cells, which has many potential in vitro applications and lays the foundation for the development of novel diagnostic tools for the detection of cancerous tissue in HNSCC and ESCC patients in vivo.

**Supplementary Information:**

The online version contains supplementary material available at 10.1186/s40170-021-00257-6.

## Background

Head and neck squamous cell carcinomas (HNSCCs) have a worldwide incidence of over 550,000 cases annually and cause over 380,000 deaths per year [1]. Despite improvements in surgical and adjuvant treatment options including laser- and robotic-based surgery [2], monoclonal antibody therapies targeting EGFR and PD-1, and molecular subtyping options [3, 4], the 5-year survival rate for HNSCCs remains below 50% [1]. Thus, the clinical outcome of HNSCC patients is persistently poor in comparison to other tumour entities and further therapy improvements are in high demand.

The standard treatment procedure for HNSCC patients consists of complete tumour resection, while preserving functionally essential anatomical structures. Advanced stages of the disease usually require a combination of surgery with irradiation and/or chemotherapeutic drugs such as 5-fluorouracil and cisplatin*.* For the surgical removal of the tumour, a resection margin of minimum 5 mm is recommended, and incomplete tumour removal is associated with worsened survival rates [5]. Rates of positive surgical margins (PSMs), i.e. incomplete tumour resection, range from 15 to 30% in head and neck cancer patients [6, 7]. Incomplete tumour resection can result from topological constraints and residual tumour cells remaining undetected during surgery. That is why further improving surgical resection techniques is an important clinical aim in addition to better diagnostic tools to support therapy planning by distinguishing cancerous tissue from healthy tissue.

One of the main characteristics of cancer cells is their highly proliferative nature, which requires them to adapt their metabolism to produce the necessary energy and biomass to create new progeny [8]. Otto Warburg observed that cancer cells, even in the presence of oxygen, preferentially metabolise glucose through “aerobic” glycolysis instead of oxidative phosphorylation [9, 10]. This allows cancer cells to survive and proliferate in both aerobic and anaerobic conditions, which is especially relevant in solid tumours, comprised of regions of high and low oxygenation. Additionally, aerobic glycolysis indirectly provides metabolites to produce cellular building blocks, such as fatty acids and non-essential amino acids [8]. However, glycolysis results in the formation of large amounts of lactate, mostly a waste product, which cancer cells extrude at higher rates than normal cells by upregulating acid extruding transporters, e.g. NHE1 and monocarboxylate transporters [11]. Through this mechanism, cancer cells maintain a higher intracellular pH (pH_i_) than normal cells, which contributes to efficient proliferation, migration, and metabolism [12]. As a further result, the extracellular pH (pH_e_) of cancer cells in tumours is measurably lower compared to non-malignant tissues with average ranges of pH 6.8–7.0 for tumour vs. 7.4 for normal cells [13, 14].

Both oxygen consumption rate (OCR) and extracellular acidification rate (ECAR) are indicators of cellular metabolic activity. They are usually determined by quantifying decreases in oxygen concentration and pH of the medium surrounding the cells over time, respectively. However, in order to distinguish cancerous from healthy tissue, a diagnostic tool must provide spatial resolution of OCR and ECAR within a tissue. In contrast to electrode-based or soluble sensor-based tools, immobilised pH- or O_2_-sensitive fluorophores are suitable options for such an application. The VisiSens TD system (PreSens GmbH) in combination with pH- and O_2_-sensitive sensor foils facilitates spatiotemporal monitoring of pH and O_2_ concentrations, both in vitro and in vivo [15–18]. In the present study, we developed a device based on the existing VisiSens TD camera system [19, 20] that allows *s*patio*t*emporal p*O*_*2*_ and *p*H *q*uantification (STO_2_P-Q). Our results show that the newly developed measurement system can detect differences in OCR and ECAR between HNSCC/ oesophageal squamous cell carcinomas (ESCC) cell lines and normal cells, which lays the foundation for the development of diagnostic tools based on the VisiSens technology.

## Methods

### Plasmid generation

mCherry was PCR amplified and cloned into the pCAG-3SIP plasmid using EcoRI and NheI restrictions sites. Additionally, the promotor was changed from CMV to EF1.

### Cell culture and generation of transgenic cell lines

FaDu, Kyse30, Cal33, and Cal27 were purchased from DSMZ (Braunschweig, Germany), HNEpC from PromoCell (Heidelberg, Germany). HFF-1 cells were initially provided by Prof. Markus Meissner and were subsequently purchased from ATCC (Manassas, USA) (SCRC-1041). FaDu cells were initially isolated from squamous cell carcinoma of the hypopharynx [21]. Kyse30 cells are derived from a well-differentiated invasive oesophageal squamous cell carcinoma [22]. Cal27 were established from a poorly differentiated squamous cell carcinoma of the tongue, and Cal33 from a moderately differentiated squamous cell carcinoma of the tongue [23]. HNEpC are primary nasal epithelial cells isolated from healthy individuals, HFF-1 are fibroblasts isolated from the foreskins of new-born human males. All cell lines were cultured in T25 or T75 cell culture flasks (Sarstedt AG, Nümbrecht, Germany) at 37 °C, 5% CO_2_, and 100% humidity. Cells were grown to 70–90% confluence and then split 1:3 to 1:10, one to three times a week. FaDu, Cal33, Cal27, and HFF were cultured in DMEM (Gibco, Dublin, Ireland) supplemented with 10% FBS (FBS Superior, Sigma Aldrich, St. Louis, USA) and 1% penicillin/streptomycin (final concentration 100 μg/ml, Gibco, Dublin, Ireland). Kyse30 cells were cultured in RPMI 1640 with l-glutamine (Gibco, Dublin, Ireland) supplemented with 10% FCS and 1% penicillin/streptomycin. HNEpC were cultured in Airway Epithelial Medium containing Supplement Mix according to the manufacturer’s instructions (PromoCell, Heidelberg, Germany).

Kyse30 and FaDu cells were transfected with a pCAG-EF1-mCherry expression plasmid using the MaTra Kit (PromoCell, Heidelberg, Germany) according to manufacturer’s instructions. Two days after transfection, transfected cells were selected by adding 1 μg/ml puromycin to the culture medium. After 2 to 3 weeks, the selected cells were resistant to puromycin and mCherry expression was verified by fluorescence microscopy.

### O_2_ and pH measurements using the newly developed device

#### Short-term measurements: standard procedure

Each separate batch of sensor foils (PreSens, Regensburg, Germany) was separately calibrated. O_2_ (SF-RPSu4-NAU-L4/W4-OIW) and pH (SF-HP5R-L4/W4-US) sensor foils were provided by PreSens, Regensburg, Germany. Calibration of O_2_ sensor foils was performed using a saturated solution of Na_2_SO_3_ in H_2_O for the 0% O_2_ calibration point and air saturated medium for the 100% O_2_ calibration point. pH calibration was performed using NaH_2_PO_4_/Na_2_HPO_4_ buffers with a buffer capacity of 40 mM, substituted with NaCl to a total ionic strength of 140 mM with six different pH values between pH 5 and pH 8.5.

To measure the oxygen consumption and extracellular acidification of individual cell lines, 4.5 × 10^4^ cells were seeded into one well of 2-well silicon inserts (Ibidi, Gräfelfing, Germany) inserted into the wells of a 6-well plate (Sarstedt AG, Nümbrecht, Germany), and cells were allowed to attach overnight. The silicon inserts were removed and 2 ml imaging medium (RPMI 1640 without Phenol Red, with l-glutamine) (Gibco, Dublin, Ireland) supplemented with 10% FCS and 1% penicillin/streptomycin added to each well. The 6-well plate was then transferred to an incubator containing the STO_2_p-Q device, placed on the frame and the lid replaced by a lid equipped with sensor foil-covered plungers. The sensor foils were equilibrated in PBS for at least 1 h before each measurement. Cells were equilibrated to the incubator’s environment for 1 h before measurement was started. To start measurements, plungers were lowered onto the cells and the VisiSensVS Software (PreSens) programmed to acquire images in 10-s intervals for 30 min (pH measurements) or 20-s intervals for 60 min (pO_2_ measurements). The exposure time was set between 400 and 600 ms, the gain was set to 3, and LED intensity set to maximum.

After measurement, cells were stained with 1 μg/ml Hoechst 33342 fluorescent DNA staining dye for 10 min. Microscopic images over the entire area of the cell layer were taken in the DAPI channel (excitation 350/50, emission 460/50) using the tile scanning function of the LASX software and a Leica DMi8 microscope (Leica, Wetzlar, Germany) with a × 10 objective (HC PL FLUOTAR 10x/0.32 PH1). Nuclei were counted as described below to normalise OCR and ECAR for cell numbers.

#### Short-term measurements: inhibitor experiments

Oligomycin (Abcam, ab141829, Cambridge, UK), 2-Deoxy-d-Glucose (2-DG) (Abcam, ab142242, Cambridge, UK), glucose (Merck, Darmstadt, Germany), and Carbonyl cyanide-4-(trifluoromethoxy)phenylhydrazone (FCCP) (Abcam, ab120081, Cambridge, UK) were diluted in DMSO or ddH_2_O according to manufacturer’s instructions. Cells were prepared according to the standard procedure described above. Drugs were added to the imaging medium and the plate incubated for 20 min on the device’s frame before starting the measurements. pO_2_ measurements were performed in 20-s intervals for 30 min, and pH measurements in 10-s intervals for 30 min. After the measurements, nuclei were stained and microscopic images acquired as described above.

### Cell landscapes

To create two-dimensional (2D) cell landscapes with defined shapes and areas, 4-well silicon inserts and 4-well silicon μ-inserts (Ibidi, Gräfelfing, Germany), respectively, were inserted into 6-well plates. 7.5 × 10^4^ cells were seeded into each well of the larger silicon inserts, and between 1 × 10^3^ and 8 × 10^3^ cells were seeded into the smaller silicon inserts. Cells were allowed to attach over night before the inserts were removed and 2 ml imaging medium was added to each well of the 6-well plate. Before measurements were started, plates were equilibrated to the incubator environment for 1 h. pH and pO_2_ were measured in 20-s intervals for 60 min, as described above.

In a second approach 200–800 FaDu or Kyse30 cells were seeded into the wells of a 6-well plate and allowed to grow into visible colonies for 7–14 days. Two millilitres of imaging medium was added to each well before microscopy, pH and pO_2_ measurements were performed as described above (20-s intervals, 60-min duration).

To create mixed 2D cell landscapes, small drops (5–20 μl) of cell suspensions of both HFF and FaDu (pCAG-EF1-mCherry) or Kyse30 (pCAG-EF1-mCherry) with 1 × 10^6^–2 × 10^6^ cells/ml were pipetted into the wells of a 6-well plate to create different patterns. After a few minutes, the drops were connected using a 10-μl pipet tip. Approximately 0.5–1 ml of medium was carefully added to the edge of the well around the drops to prevent evaporation. Cells were allowed to adhere overnight, and then 2 ml of imaging medium was added to each well. Microscopic images over the entire area covered by cells were taken in phase contrast, and in the TXR channel (excitation 560/40, emission 639/75) with a × 5 or × 10 objective using the tile scanning function of the LASX software and a Leica DMi8 microscope. pH and pO_2_ measurements were performed as described above (20-s intervals, 60-min duration).

In a different approach 200–800 FaDu (pCAG-EF1-mCherry) or Kyse30 (pCAG-EF1-mCherry) cells were seeded into the wells of a 6-well plate and allowed to grow into visible colonies for 7–14 days. Subsequently, 500,000 HFF were seeded into each well containing colonies. The cells were allowed to adhere overnight and then 2 ml of imaging medium was added to each well. Microscopy, pH and pO_2_ measurements were performed as described above (20-s intervals, 60-min duration).

In a third approach, 500,000 HFF were seeded into the wells of a 6-well plate, allowed to grow for 3 days and subsequently irradiated with 35 Gy using a CIX2 cabinet irradiator (Xstrahl, Camberley, UK) equipped with a 0.5-mm Cu filter. Two days later 200–800 FaDu (pCAG-EF1-mCherry) or Kyse30 (pCAG-EF1-mCherry) cells were seeded onto the HFF cell layers and allowed to grow into visible colonies for 7–14 days. Two millilitres of imaging medium was added to each well before microscopy, and pH and pO_2_ measurements were performed as described above (20-s intervals, 60-min duration). For all colony-based experiments colony sizes were measured using Fiji [24].

#### Long-term measurements

Self-adhesive pH sensor spots (PreSens) were inserted into 96-well plates and pre-treated with 100 μl FCS for 1 h at 37 °C before cells were seeded to improve cell adhesion. For O_2_ measurements, 96-well plates with the entire bottom consisting of O_2_ sensor foil material were equilibrated with 100 μl PBS for 1 h at 37 °C. PBS and FCS, respectively, were removed and pH sensors washed with PBS once before seeding cells.

To compare the pH and pO_2_ in FaDu cell cultures depending on cell numbers, 5000, 10,000, 20,000, 40,000, and 60,000 FaDu cells were seeded into 96-well plated equipped with pH sensor spots or with the bottom consisting of O_2_ sensor foil. For each cell number, six wells were used, i.e. six technical replicates. To compare cell lines, 40,000 cells of FaDu, Kyse30, Cal33, Cal27, HFF, and HNEpC were seeded into six wells each (six technical replicates). O_2_ and pH calibration buffers were included for each separate batch of sensor foils, as described above. The 96-well plates containing the cells were sealed with a gas permeable foil (Biozym, 600295, Vienna, Austria) to prevent evaporation and left in the laminar flow cabinet for 1 h to improve equal distribution of cells in the wells. Plates were then placed on the frame and pH or O_2_ measured for 90 h in 1-h intervals.

### Extracellular flux analysis (Seahorse XFe96)

Approximately 20 h before measurement, 4 × 10^4^ cells/well were seeded into XF96 microplates in five technical replicates per cell line. One hour before the assay, culture medium was replaced by Seahorse assay medium (XF RPMI 103576-100, Agilent, Santa Clara, USA) containing 2 mM l-glutamine and 5 mM glucose (for OCR measurements). Basal OCR and ECAR, and ECAR measurements after injection of glucose (5 mM final concentration) were performed in three measurement cycles, with a mix duration of 3 min, and measurement duration of 3 min. OCR and ECAR shown here represent the basal OCR and ECAR after injection of glucose, from the third measurement cycle. Immediately after the assay was run, cell numbers were imaged in each well using Hoechst 33342 DNA staining and quantified by a multi-imager (Cytation1, BioTek Instruments, Winooski, USA). Metabolic activities, i.e. OCR and ECAR, were normalised to the respective cell number and calculated per 10^4^ cells.

### Software, data analysis, and statistics

#### OCR and ECAR calculation from short-term measurements

After the application of a noise filter and the sensor calibration using the VisiSensVS software, regions of interest (ROI) were drawn around areas that corresponded to the cell layer, and pO_2_ concentrations over time exported to a data frame using the “multi-z” tool. For pH measurements, noise filter and sensor calibration were performed using the VisiSensVS software. The images and calibration files were then imported into the VS Live Plugin (Release 0.4.7) (PreSens, Regensburg, Germany). ROI were drawn and pH values over time exported to a data frame. pH and pO_2_ were plotted using R [25] and the R package ggplot2 [26]. A linear regression was calculated for the linear range of the pH and O_2_ curves. The respective slopes of the regression lines represent the decrease of O_2_ concentration or pH over time (%O_2_/s and pH/s), thus the OCR and ECAR. To normalise for cell numbers, the slope was divided by the respective cell numbers calculated from fluorescent images of the cells’ nuclei and multiplied by a factor of 10^7^ (for OCR) and 10^4^ (for ECAR) to improve the way of representation of the data, as indicated in the respective figures. The normalised OCR and ECAR were plotted as dotplots using R and ggplot2.

#### Analysis of long-term measurements

After data acquisition, noise correction and sensor calibration were performed using the VisiSensVS software (PreSens, Regensburg, Germany). Image and calibration files were then loaded into the VDT 96-well Extractor programme (PreSens, Release 0.3.1.2). A one-point adjustment (“OPA”) was performed using an image of a plate with the same pO_2_ or pH in all wells to correct for uneven illumination of the plate. pO_2_ and pH values were then automatically exported to a CSV file. The mean and standard deviation were calculated and data were plotted as line graphs using R and ggplot2.

#### Quantification of cell numbers

Sixteen-bit greyscale images obtained by fluorescence microscopy were loaded into CellProfiler [27] and nuclei detected using the module “IdentifyPrimaryObjects” with advanced settings that were adjusted according to fluorescence intensity, nuclear size, and density of cells.

#### Image processing and figure preparation

For micrographs shown in this paper, contrast was adjusted linearly and scale bars and labels added using Fiji [24]. Figures were assembled and overlays drawn using Inkscape [28].

#### Statistical analyses

All statistical analyses were performed using R and the package rstatix. To determine differences between different groups, one-way ANOVA tests with post hoc Tukey HSD were performed. Differences were considered to be statistically significant when *p* ≤ 0.05.

## Results

### Fluorescence-based spatiotemporal measurement of O_2_ consumption and extracellular pH in HNSCC cells

**Fig. 1:**
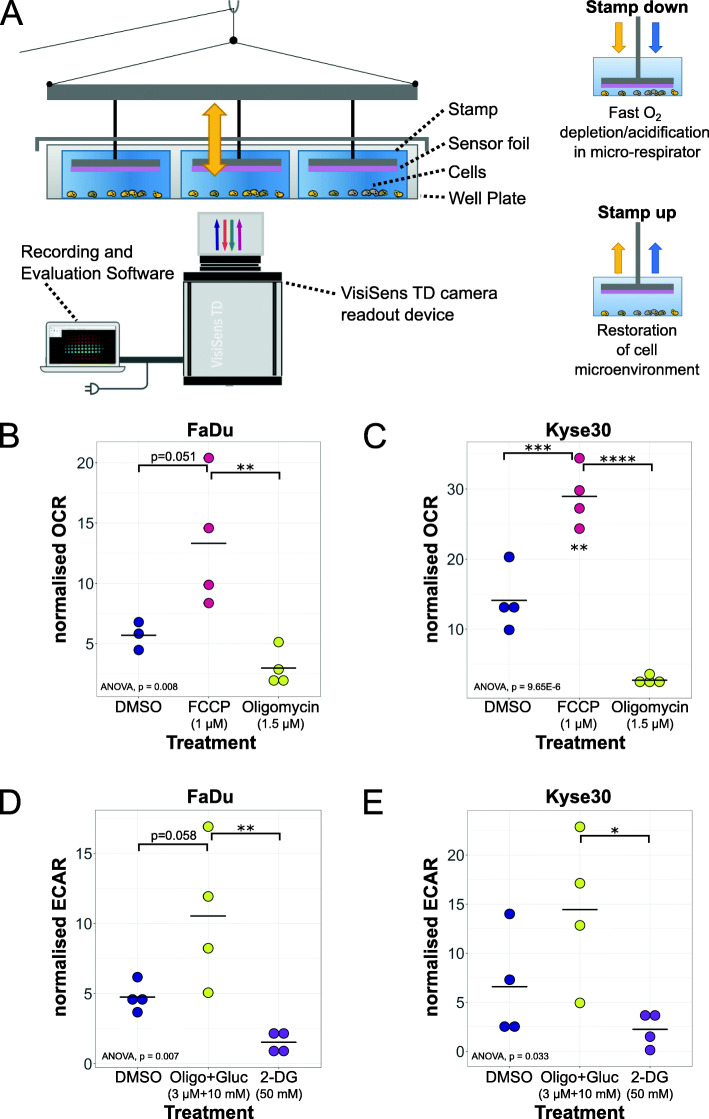
Introduction of the STO_2_p-Q method. **a** Schematic representation of STO_2_p-Q for the measurement of OCR and ECAR. Through lowering of sensor foil-equipped stamps, a micro-respirator is generated facilitating fast measurements of OCR and ECAR. OCR measured using STO_2_p-Q of FaDu (**b**) and Kyse30 (**c**) cells treated with 1.5 μM oligomycin (Oligo) and 1 μM FCCP and DMSO (1:10,000). OCR was normalised by dividing the OCR (decrease of pO_2_ (%)/time (s)) by the cell number and multiplying with a factor of 10^7^ (normalised OCR). *n* = 4, from independent experiments. ECAR measured using STO_2_p-Q of FaDu (**d**) and Kyse30 (**e**) cells treated with 3 μM oligomycin and 10 mM glucose (Oligo+Gluc) and 50 mM 2-Deoxy-d-Glucose (2-DG) and DMSO (1:5000). ECAR was normalised by dividing the ECAR (decrease of mpH/time (min)) by the cell number and multiplying with a factor of 10^4^ (normalised ECAR). *n* = 5, from independent experiments. Data was plotted as dot plots, horizontal black line indicates the mean. Statistical analysis was performed to compare different treatment groups (one-way ANOVA with post hoc Tukey HSD). *p < 0.05, **p < 0.01, ***p < 0.001, ****p < 0.0001

To measure O_2_ consumption and extracellular acidification in cell cultures, we developed a device for the spatiotemporal pO_2_ and pH quantification in adherent cell cultures in a 6-well format (STO_2_p-Q). STO_2_p-Q enables a temporary reduction of the media volume surrounding the cells, creating a micro-respirator and subsequent measurement of the pO_2_ or pH in the cellular microenvironment by lowering sensor foil-equipped plungers onto the cells in each well simultaneously. The plungers consist of metal discs (weight 4 g), connected to strings that are routed through the lid of a 6-well plate and attached to a plastic lid that can be lowered and lifted through an attached string routed through a loop and then through the door of a cell culture incubator. This facilitates the operation of STO_2_p-Q in an incubator without disturbing the environmental conditions, i.e. temperature and CO_2_ concentration. Within the incubator, the measurement plate is placed on a frame that facilitates the excitation and detection of the fluorescence from below using the VisiSens TD camera readout device. LEDs facilitating excitation were attached to the frame (Fig. 1a).

To test if STO_2_p-Q enables accurate measurements of OCR and ECAR, effects of known disruptors of mitochondrial respiration and glycolysis, respectively, were measured in a HNSCC and ESCC cell line, respectively. FaDu and Kyse30 cells were seeded into one well of 2-well silicone inserts, inserted into a 6-well plate, and allowed to attach and form a confluent layer. After the addition of the inhibitors, pO_2_ and pH measurements were performed as described in the methods section. FCCP is an uncoupler of mitochondrial oxidative phosphorylation and is expected to increase cellular oxygen consumption, while oligomycin, an inhibitor of H^+^ ATP-synthase, inhibits mitochondrial respiration and thus is expected to decrease cellular oxygen consumption. In FaDu cells, addition of 1 μM FCCP increased the cells’ oxygen consumption compared to the DMSO control, while the addition of 1.5 μM oligomycin decreased the OCR significantly compared to the FCCP treatment (Fig. 1b). In Kyse30 cells, the effect of the inhibitors was more pronounced, with FCCP significantly increasing and oligomycin significantly decreasing the OCR compared to the DMSO control (Fig. 1c).

To manipulate the glycolysis rate and thus lactate production and subsequent extracellular acidification, 2-DG and oligomycin in combination with glucose were used. 2-DG is a glucose analogue that competes with glucose for phosphoglucoisomerase but cannot be metabolised, thus inhibiting glycolysis. Oligomycin blocks mitochondrial respiration and in the presence of glucose is expected to increase the ECAR. In FaDu cells the addition of 3 μM oligomycin and 10 mM glucose caused an increase in the ECAR, while 50 mM 2-DG resulted in a lower ECAR compared to the DMSO control, however not statistically significant (Fig. 1d). In Kyse30 cells, the measurements were more variable with the only statistically significant difference observed between cells treated with 2-DG and oligomycin and glucose, with oligomycin and glucose showing a significantly higher ECAR (Fig. 1e).

The results demonstrate that STO_2_p-Q in combination with the here established data analysis pipeline is able to distinguish differences in OCRs and ECARs between cells treated with different compounds.

### Differences in ECAR and OCR between HNSCC cell lines and healthy cells

**Fig. 2:**
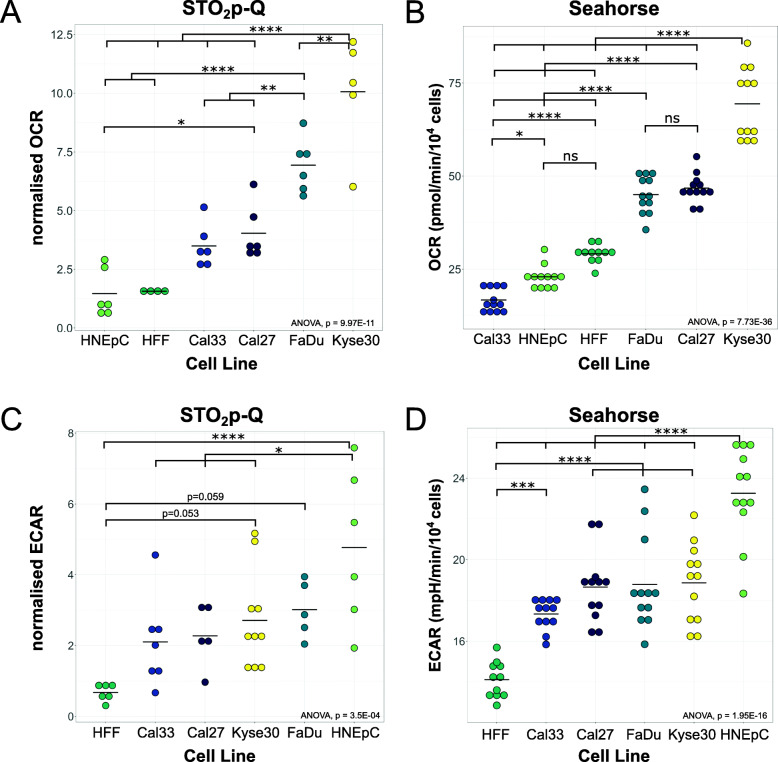
OCR and ECAR measurements in HNSCC and normal cells. **a** OCR measured using STO_2_p-Q of FaDu, Kyse30, Cal33, Cal27, HNEpC, and HFF cells. OCR was normalised by dividing the OCR (decrease of pO_2_ (%)/time (s)) by the cell number and multiplying with a factor of 10^7^ (normalised OCR). FaDu, *n* = 6; Kyse30, *n* = 5; Cal33, *n* = 6; Cal27, *n* = 6; HNEpC, *n* = 6; and HFF, *n* = 4, from independent experiments. **b** OCR measured using extracellular flux analysis (Seahorse). Shown are basal OCR. *n* ≥ 12, from two independent experiments. **c** ECAR measured using STO_2_p-Q of FaDu, Kyse30, Cal33, Cal27, HNEpC, and HFF cells. ECAR was normalised by dividing the ECAR (decrease of mpH/time (min)) by the cell number and multiplying with a factor of 10^4^ (normalised ECAR). *n* ≥ 5, from independent experiments. **d** ECAR measured using extracellular flux analysis (Seahorse). Shown is the ECAR after injection of 5 mM glucose. *n* = 12, from two independent experiments. Data was plotted as dot plots; horizontal black line indicates the mean. Statistical analysis was performed to compare different cell lines (one-way ANOVA with post hoc Tukey HSD). *p < 0.05, **p < 0.01, ***p < 0.001, ****p < 0.0001

One of our aims was to develop a method to distinguish healthy tissue from cancerous tissue in patients with HNSCCs. As a first step, we explored potential differences in OCR and ECAR between HNSCC/ESCC cells and normal cells in vitro. In order to identify potential differences, three established HNSCC cell lines (FaDu, Cal33, Cal27), one ESCC cell line (Kyse30), and two normal cell lines (human foreskin fibroblasts, HFF; primary human nasal epithelial cells, HNEpC) were measured using STO_2_p-Q equipped with O_2_ or pH sensor foils as described. In Kyse30 cells, the measured OCR was significantly higher than in all other cell lines. FaDu cells displayed the second highest OCR, differing significantly from Cal33, Cal27 and the two normal cell lines, HFF and HNEpC, both of which showed the lowest OCR. The OCR of Cal33 and Cal27 did not differ significantly from HFF and HNEpC (Fig. 2a). To verify our results obtained by STO_2_p-Q, we performed OCR measurements in all cell lines via the established method of extracellular flux analysis using the Seahorse technology. The results obtained from the Seahorse measurements reflected the results obtained by our method in that Kyse30 displayed the highest basal OCR, followed by FaDu and Cal27, and showing significantly higher OCR than HFF and HNEpC cells. However, in the Seahorse measurements Cal33 showed a significantly lower OCR than HFF and HNEpC (Fig. 2b).

Using STO_2_p-Q, the pH measurements showed a significantly higher ECAR in HNEpCs compared to all other cell lines except FaDu, while HFFs exhibited the lowest ECAR. Differences between HFF and HNSCC cell lines were not statistically significant; however, a tendency for higher OCR, especially in Kyse30 and FaDu, compared to HFF was detectable (Fig. 2c). The results from the Seahorse measurement confirmed a low ECAR (in the presence of 5 mM glucose) in HFFs, which differed significantly from all other cell lines, while there was no significant difference between the HNSCC/ESCC cell lines (Fig. 2d), comparable to the results obtained with STO_2_p-Q.

### Spatial discrimination of different cell lines based on O_2_ consumption

**Fig. 3:**
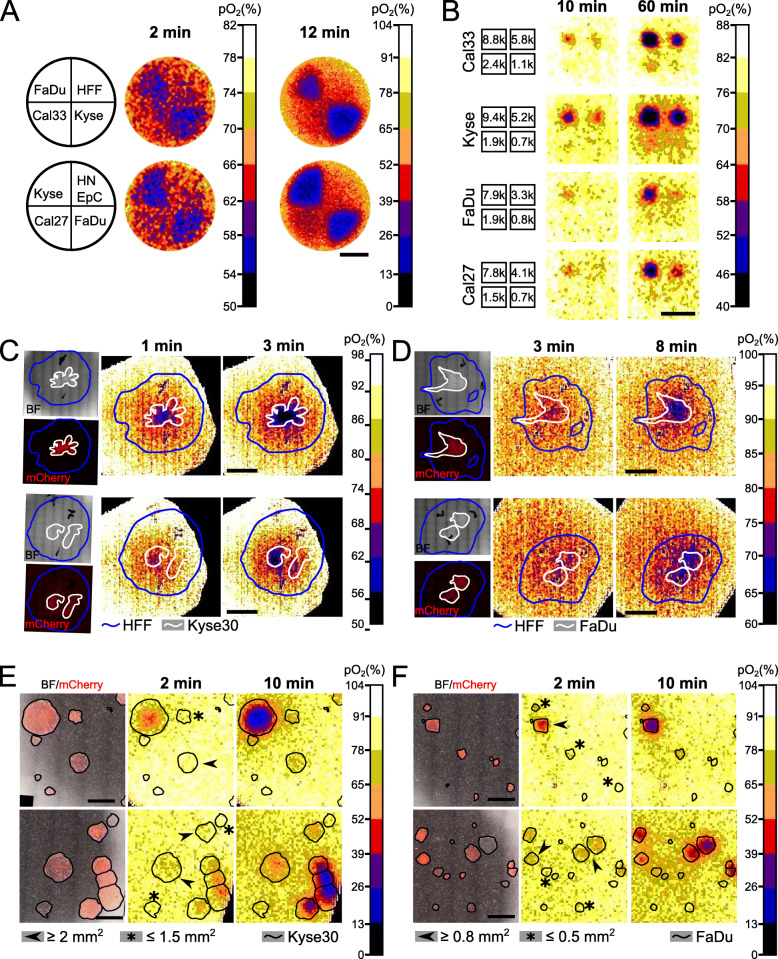
Spatial resolution of cell types based on O_2_ consumption measured using STO_2_p-Q. **a** 7.5 × 10^4^ cells (FaDu, Kyse30, Cal33, Cal27, HNEpC, HFF) were seeded into 4-well silicon inserts, as shown on the left. Heat maps represent pO_2_ after 2 and 12 min, measured using STO_2_p-Q. Noise filter was applied with a smoothing factor of 2 (2-min timepoint). **b** Different cell numbers of FaDu, Kyse30, Cal33, and Cal27 were seeded into 4-well silicon μ-inserts (k, thousand; e.g. 8.8k = 8800 cells). Heat maps represent pO_2_ after 10 and 60 min. Noise filter was applied with smoothing factor of 2. Scale bar, 5 mm. HFF and Kyse30 (**c**) or FaDu (**d**) cells expressing mCherry were seeded into a 6-well plate to create different 2D-landscapes. pO_2_ heat maps and microscopic images were overlaid, and the borders of cell layers outlined in white (Kyse30/FaDu) and blue (HFF) based on phase contrast images (BF, bight field) and mCherry signal. Heat maps represent pO_2_ after 1 and 3 min (**c**) or after 3 and 8 min (**d**). Scale bars, 5 mm. HFF cells were seeded on Kyse30 (**e**) or FaDu (**f**) colonies expressing mCherry growing in 6-well plates. pO_2_ heat maps and microscopic images were overlaid, and the Kyse30/FaDu colonies outlined in black based on phase contrast images (BF, bight field) and mCherry signal. Heat maps represent pO_2_ after 2 and 10 min. Scale bars, 2.5 mm. For all measurements, representative images are shown

Detection of the spatial distribution of tumour cells and healthy cells is particularly relevant for clinical applications. To test the ability of STO_2_p-Q to identify areas containing tumour cells and to define the detection limit and resolution, different 2D-patterns of cells (“landscapes”) were created. O_2_ concentrations in the cellular landscapes were measured using the device equipped with O_2_ sensor foils. First, HNSCC/ESCC cells and normal cells were seeded into 4-well silicon inserts with a growth area of 0.35 cm^2^/well and with each well containing a different cell type (Fig. 3a). After removal of the silicon inserts, measurements were performed. The wells containing Kyse30 and FaDu cells could be identified after 2 min based on the O_2_ heat maps and after 12 min the outlines of the areas containing FaDu and Kyse30 cells were clearly detectable (Fig. 3a). Areas containing Kyse30 cells were characterised by the lowest O_2_ concentration, followed by FaDu, Cal27, Cal33, HNEpC, and HFF, reflecting the previous results from measurements of individual cell lines (see Figs. 2a and 3a).

Next, we were interested in defining an approximate detection limit for HNSCC/ESCC cell densities based on their OCR. Therefore, different cell numbers between 1000 and 8000 cells of FaDu, Kyse30, Cal22, and Cal27 were seeded into 4-well silicon μ-inserts with a growth area of 0.03 cm^2^/well. O_2_ concentrations were measured after removal of the silicon insert. Exact cell numbers were determined subsequently by fluorescence microscopy. After 10 min, the wells containing the highest cell numbers could be visualised in all cell lines, with the strongest signal, i.e., lowest O_2_ concentration, in Kyse30 cells. After 60 min, the two highest cell densities (i.e. from 7800 to 9400 cells and 3300 to 5800 cells, respectively) were detectable in all cell lines, and a weak signal was visible in most cell lines for the second lowest cell density (i.e. 1500 to 2400 cells) (Fig. 3b). This indicates that the detection limit for the HNSCC cells used in this study is at approximately 2000 cells growing in an area of 0.03 cm^2^, which translates to a cell density of approximately 66,000 cells/cm^2^.

In an approach to determine the minimum size of a cell colony resolvable by STO_2_p-Q, FaDu and Kyse30 cells were seeded into 6-well plates at low density and allowed to grow into colonies of different sizes. The cells in Kyse30 colonies were evenly spread out and forming a two-dimensional layer, while FaDu colonies were growing in dense and packed colonies where cells appeared to grow in multiple layers. Kye30 colonies were detectable after 10 min down to a size of 1 mm^2^ (Additional file 1, A). FaDu colonies could be detected down to a size of 0.6 mm^2^ within 2 min (Additional file 1, B).

To create a scenario that closely resembles an in vivo situation, HNSCC/ESCC cells and normal cells were seeded together in 2D-landscapes. As Kyse30 and FaDu cells showed the highest OCR in previous measurements, they were used in this experiment, together with HFF, which showed the lowest OCR (see Figs. 2a and 3a). The respective tumour cells stably expressed a cytosolic mCherry fluorescent protein that enabled us to identify the cells by fluorescence microscopy prior to the O_2_ measurements. After the measurements, the outline of tumour and normal cell areas, obtained from phase contrast and fluorescence micrographs, was overlaid with the O_2_ heat maps to mark the areas occupied by cells. Areas with Kyse30 cells within a layer of HFF were first detectable after 1 min of measurement. After 3 min, the areas containing the tumour cells were more clearly identifiable based on O_2_ heat maps. However, areas with an apparently lower cell density or smaller extensions of the tumour cell layer into the HFF layer were not represented in the O_2_ heat maps (Fig. 3c). In the landscapes with FaDu cells, areas containing tumour cells started being visible in the O_2_ heat maps after 3 min and were more clearly detectable after 8 min. Similar to the landscapes with Kyse30 cells, areas with lower cell densities and extensions were not resolved (Fig. 3d). The intensity of the signal for FaDu cells was lower than in Kyse30 cells and the time until detection of a signal was longer, which is in accordance with the lower OCR measured in FaDu cells compared to Kyse30 in previous experiments (Fig. 2a).

In an alternative approach, mCherry expressing FaDu or Kyse30 cells were seeded into 6-well plates at low density and allowed to form colonies. Subsequently, HFF cells were seeded into the wells and arranged themselves around the colonies to form a single layer. Areas containing Kyse30 colonies larger than 2 mm^2^ could be distinguished from HFF cells between 2 and 10 min of measurement (Fig. 3e), while FaDu colonies with a size between 0.5 and 0.8 mm^2^ were resolvable (Fig. 3f), in accordance with the results from the measurements of individual colonies. Alternatively, HFF cells were seeded first and FaDu and Kyse30 colonies allowed to grow on the layer of HFF cells. While Kyse30 cells formed colonies with low cell densities that could only be detected after approximately 30 to 50 min (Additional file 1, C), FaDu cells formed dense colonies that were detectable between 2 and 10 min (Additional file 1, D).

With these experiments, we demonstrated that it is feasible to spatially distinguish cell types based on their two-dimensional O_2_ consumption profiles using STO_2_p-Q and determined the approximate limit of resolution.

### Spatial discrimination of different cell lines based on extracellular acidification

**Fig. 4:**
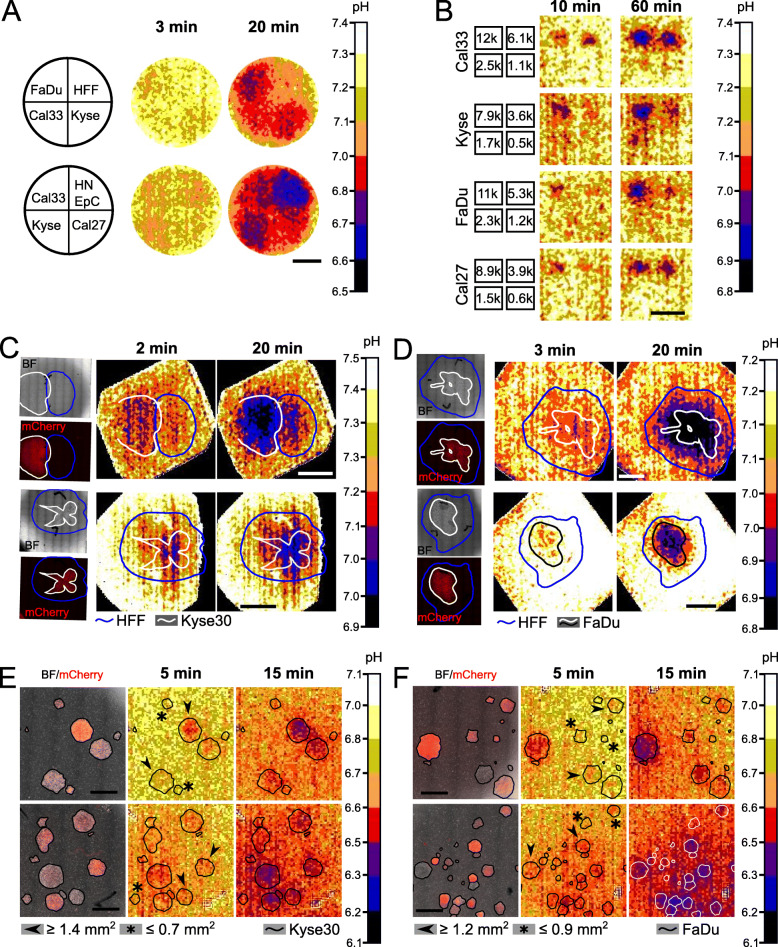
Spatial resolution of cell types based on extracellular acidification measured using STO_2_p-Q. **a** 7.5 × 10^5^ cells (FaDu, Kyse30, Cal33, Cal27, HNEpC, HFF) were seeded into 4-well silicon inserts, as shown on the left. Heat maps represent pH after 3 and 20 min, measured using STO_2_p-Q. **b** Different cell numbers of FaDu, Kyse30, Cal33, and Cal27 were seeded into 4-well silicon μ-inserts (k, thousand, e.g. 6.1k = 6100 cells). Heat maps represent pH after 10 and 60 min. HFF and Kyse30 (**c**) or FaDu (**d**) cells expressing mCherry were seeded into a 6-well plate to create different 2D-landscapes. pH heat maps and microscopic images were overlaid, and the borders of cell layers outlined in white (Kyse30/FaDu) and blue (HFF) based on phase contrast images (BF) and mCherry signal. Heat maps represent pO_2_ after 2 and 20 min (**c**) or 3 and 20 min (**d**). Scale bars, 5 mm. **d** HFF and FaDu cells were seeded into a 6-well plate to create different 2D-landscapes. pO_2_ heat maps and microscopic images were overlaid, and the borders of cell layers outlined in white/black (FaDu) and blue (HFF) based on phase contrast (BF) and mCherry images. Heat maps represent pO_2_ after 3 and 20 min. Scale bars, 5 mm. HFF cells were seeded on Kyse30 (**e**) or FaDu (**f**) colonies expressing mCherry growing in 6-well plates. pH heat maps and microscopic images were overlaid, and the Kyse30/FaDu colonies outlined in black or white based on phase contrast images (BF, bight field) and mCherry signal. Heat maps represent pH after 5 and 15 min. Scale bars, 2.5 mm. Noise filter was applied with a smoothing factor of 2 for **a** to **d**. For all measurements, representative images are shown

To test the ability of STO_2_p-Q to spatially distinguish different cell types based on extracellular acidification, we repeated the experiments described above, measuring pH instead of O_2_ concentrations. When seeded into 4-well silicon inserts, areas containing cells were first detectable in the pH heat map after 3 min. After 20 min, areas containing HNEpC cells exhibited the lowest pH (Fig. 4a). This is in accordance with previously measured ECARs for HNEpCs and HFFs, representing the cells with the highest and lowest ECAR, respectively (Fig. 2c).

Next, to determine the detection limit of the pH sensors for tumour cells, different cell numbers were seeded into smaller 4-well silicon inserts. After 10 min, wells containing the highest cell numbers (7900–12,000 cells) could be clearly distinguished in the pH heat map. After 60 min, areas containing the second highest cell numbers (3600–6100 cells) were also clearly identifiable. In Kyse30 and Cal33, a weak signal was identified in wells with the second lowest cell number (1700 and 2500 cells, respectively (Fig. 4b)). Based on these results, the detection limit for pH measurements is in the range of 2000 and 6000 cells per 0.03 cm^2^ or between 66,000 and 200,000 cells/cm^2^.

Individual Kyse30 colonies could be detected down to a size of 1.3 mm^2^ within 10 min (Additional file 2, A), and FaDu colonies down to a size of 0.5 mm^2^ (Additional file 2, B), similar to the resolution limit of pO_2_ measurements.

Similar to the experiments with the oxygen sensor foils described above, HFFs and either Kyse30 or FaDu cells were seeded to create cellular landscapes. Areas containing Kyse30 cells started exhibiting a lower pH compared to the surrounding areas after 2 min of measurement. After 20 min those areas were clearly identifiable, however, similar to the oxygen heat maps, smaller extensions of the tumour cell area were not resolved (Fig. 4c). The landscapes with FaDu cells and HFF behaved similarly, with a decrease in pH detectable in the areas containing FaDu cells after 3 min instead of 2 min in Kyse30 cells (Fig. 4d).

Kyse30 colonies over 1.4 mm^2^ within a layer of HFF cells were detectable between 5 and 15 min of pH measurements (Fig. 4e), while FaDu colonies were detectable at a minimum size of 1.2 mm^2^ (Fig. 4f). FaDu colonies growing on top of HFF cells could be visualised down to a size of 0.6 mm^2^ (Additional file 2, D), while Kyse30 colonies had a lower cell density and individual colonies were not clearly detectable based on pH measurements (Additional file 2, C).

These experiments showed that we could spatially distinguish different cell types based on their extracellular acidification using STO_2_p-Q and defined an approximate resolution limit.

### Long-term OCR and ECAR measurements in HNSCC and normal cells

In the experiments described above, short-term measurements with STO_2_p-Q were used to quantify differences in the cell’s OCR and ECAR. However, we were also interested in investigating how cells consumed oxygen and acidified their environment in long-term culture conditions. For this purpose, 96-well plates with the bottom consisting of oxygen sensor foil material and pH sensor foil spots glued into the bottom of the wells, respectively, were used. The cells were directly seeded onto the sensor foils, which allows for monitoring of the direct cellular microenvironment. Plates were placed onto the frame also used for STO_2_p-Q, and measurements performed using the same camera system and software. Cells were allowed to grow for 90 h, during which the oxygen or pH were monitored every 60 min.

**Fig. 5:**
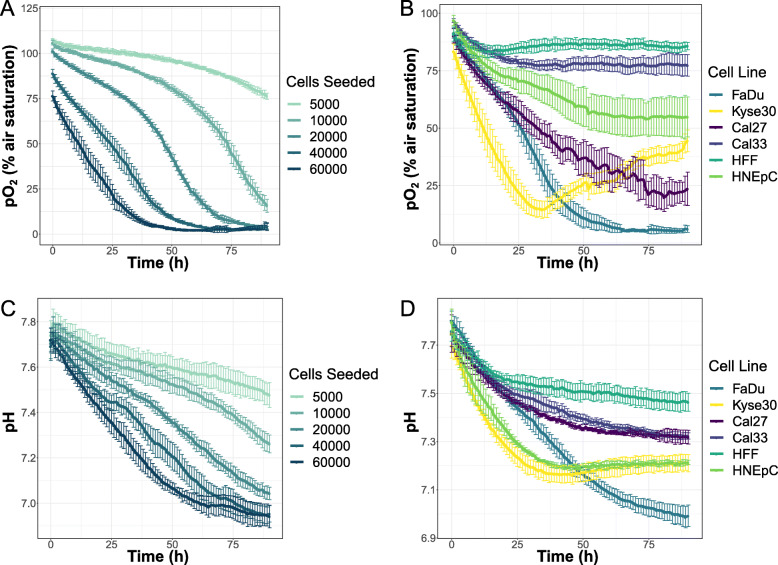
Long-term pH and pO_2_ measurements. **a** pO_2_ (in % air saturation) in FaDu cell cultures with different cell numbers over 90 h measured in 96-well plates with cells growing on O_2_ sensor foils. **b** pO_2_ (in % air saturation) in FaDu, Kyse30, Cal33, Cal27, HNEpC, and HFF cell cultures over 90 h measured in 96-well plates with cells growing on O_2_ sensor foils. **c** pH in FaDu cell cultures with different cell numbers over 90 h measured in 96-well plates with cells growing on pH sensor foil spots. **d** pH in FaDu, Kyse30, Cal33, Cal27, HNEpC, and HFF cell cultures over 90 h measured in 96-well plates with cells growing on pH sensor foil spots. All line graphs depict mean values ± standard deviation from six wells each. Plots show a representative measurement from *n* = 3 independent experiments

First, different numbers of FaDu cells were seeded and measurements were started 5 h later. The O_2_ concentration curves reflected the number of cells seeded, in that the higher the cell number, the faster the oxygen consumption (Fig. 5a).

Next, 40,000 cells of four HNSCC/ESCC cell lines (FaDu, Kyse30, Cal27, Cal33) and two normal cell lines (HFF, HNEpC) were seeded and measurements started after 5 h. Differences between cell lines became most pronounced after 25 h of measurement, with HFF cells showing the lowest oxygen consumption and FaDu and Kyse30 cells consuming oxygen faster than the other cell lines (Fig. 5b).

For pH measurements, different numbers of FaDu cells were seeded and measurements started after 1 h. The course of the pH values over time closely resembled the course of oxygen concentrations for the respective cell numbers, with high cell numbers showing a steep decrease in pH while curves for low cell numbers remained flatter (Fig. 5c).

When comparing different cell lines, pH curves of Kyse30 and HNEpC showed a similar course, representing the cell lines with the fastest acidification. FaDu cells exhibited the strongest acidification overall, while HFF showed the slowest decrease in pH (Fig. 5d).

This measurement setup allowed us to monitor the pH and oxygen concentration in the direct cellular environment over several days without interference. Furthermore, decreases in both O_2_ concentration and pH correlated with cell numbers and cell type.

## Discussion

While several different techniques exist to measure OCRs and ECARs of cells [29], they do not support easy spatial measurements. Electrode-based measurement devices rely on either moving the electrode between different localisations [30] or using multiple electrodes to gain spatial information, which limits both the spatial and temporal resolution. Fluorescence- or luminescence-based oxygen and pH measurement sensors are available in soluble forms or as immobilised sensors, as in the Seahorse system that is commonly used for the determination of OCR and ECAR [31–35]. While the Seahorse system is highly automated and thus convenient, it does not allow for spatially resolved OCR and ECAR measurements.

In this study, we describe the development and application of a newly developed device, which we have termed STO_2_p-Q referring to spatiotemporal O_2_ and pH quantification. We have used the STO_2_p-Q device to measure OCR and ECAR in HNSCC cell lines and normal cells, and found that some of the HNSCC/ESCC cell lines, especially FaDu and Kyse30 cells, consumed more oxygen than the two normal fibroblast and epithelial cell lines, which was confirmed by independent measurements with the Seahorse system. The Seahorse measurements also provided absolute OCR and ECAR for the different cell lines, which STO_2_p-Q is currently not suitable for as the media volume in the micro-respirator created by lowering the stamps is unknown. The relative differences in OCR enabled the spatial distinction of the two commonly used HNSCC/ESCC cell lines FaDu and Kyse30 from fibroblasts in a co-culture approach based on their oxygen consumption. Similarly, FaDu and Kyse30 cells could be spatially distinguished from HFFs based on their extracellular pH. This lays the foundation for further in vitro and in vivo applications, where a spatial resolution of O_2_ concentrations and pH in the cellular microenvironment is of importance.

In our experiments, tumour cell colonies down to a size of 0.5 mm^2^ could be resolved, depending on cell type and density of formed colonies. A limiting factor in gaining higher resolutions might be the rate at which O_2_ concentrations or pH can be restored in the direct cellular microenvironment through diffusion, thus preventing detection of O_2_ consumption and acidification by the sensor.

We are currently developing an automated, wireless, and programmable device that will allow for repeated short-term measurements over longer periods and for a more controlled cellular microenvironment. Diffusion could be minimised by decreasing the space between cell surface and sensor foil using stamps with a heavier weight. However, a balance between minimal diffusion and preventing damage to the cells would have to be warranted. By redesigning the stamps to have a small rim, potential horizontal diffusion at the sensor borders could be prevented. This could also facilitate the determination of the media volume in the micro-respirator, thus enabling absolute OCR and ECAR measurements and potentially improving variability between experiments. Furthermore, the resolution was limited by the camera setup used in this study. In future experiments, we will use detection cameras equipped with higher resolution objectives and thus attempt to further improve the detection limit.

The future device will also facilitate tracking changes in cell populations over time, as multiple sequences of measurements can be programmed and a sterile environment can be ensured. Combining STO_2_p-Q with a suitable excitation source and camera, a simultaneous detection of O_2_ or pH and fluorescent proteins expressed by certain cell populations is technically feasible. This would facilitate tracking of different cell populations over time and eliminate the requirement for removing the cells from the incubator to perform fluorescence microscopy between measurements.

Another application of the VisiSens technology we described in this study is long-term measurements in a 96-well format. However, these measurements do not allow for a direct determination of OCR and ECAR and comparisons of different cell lines, as cells continue to proliferate over the long measurement periods and would confound the data. However, long-term measurements are valuable for tracking changes within cell cultures in the direct cellular microenvironment over time without disturbances, which is especially important for cell lines sensitive to changes in pH or O_2_ concentrations.

In a clinical setting, future enhancement of the current technology could help identify residual cancerous tissue in patients in an intraoperative setting. Our in vitro measurements showed that the technology is capable of detecting differences in O_2_ consumption and extracellular pH between normal cells and some HNSCC/ESCC cell lines, which is a prerequisite for distinguishing healthy from cancerous tissue also in vivo. As local recurrences in HNSCC are very frequent, there is an urgent need for improving both early detection of cancer and for superior techniques to detect cancerous tissue during resection of the primary tumour. A variety of novel approaches has been discussed such as intraoperative molecular imaging [6], or specimen mapping by near-infrared fluorescence [36]. Especially real time, intraoperative imaging of tumour cells is promising to obtain additional visual information and to avoid residual disease. A modified version of the VisiSens system has been used previously to investigate pO_2_ and pH gradients in chronic and acute wounds [16] and in postoperative wounds after radiation therapy [37]. These studies have used transparent flexible sensor foils in combination with a handheld device for excitation of the sensor foils and acquisition of O_2_ concentrations and pH. In accessible areas of the head and neck, this setup could be used to assess cancerous and surrounding tissues before, during, and after surgery to identify the specific outlines of the tumour. Accordingly, we are currently preparing a study to investigate if cancerous tissue can be detected based on O_2_ consumption and pH in vivo. One of the main advantages of a setup using sensor foils in combination with a handheld device is that no treatment, e.g. labelling, of the tissue would be necessary and that the measurements can be performed in a timeframe of minutes [37]. The applicability of sensor foils on uneven tissue and in the presence of liquids, e.g. saliva and blood, will have to be assessed in future experiments, as those could be potential obstacles to clear measurements. Similarly, the effect of food, medication, etc. on the variability of pH measurements in the oral cavity might represent a challenge.

Another clinical application, where pH and pO_2_ are of major importance, is radiation therapy as a common treatment in neck and head cancer. Despite benefits with respect to disease control, irradiation may show interference with wound repair processes. Sufficient oxygenation is mandatory for essential molecular healing processes like cell proliferation and protein synthesis [17]. Chronic wounds in irradiated patients show hypoxic characteristics [37], qualifying pO_2_ as a major parameter in monitoring wound healing progresses after surgical interventions. Furthermore, damage of healthy skin through radiotherapy is reflected by an altered pH and pO_2_ compared to non-irradiated skin [37].

Therefore, pO_2_ and pH represent metabolic parameters of clinical and prognostic value and a device that allows for a standardised and easy measurement of these parameters would represent a considerable benefit for patients in numerous different settings.

## Conclusions

In this study, we show the development of a device, the STO_2_p-Q, to spatiotemporally measure OCR and ECAR in cells. Using STO_2_p-Q, we were able to measure differences in OCR between HNSCC/ESCC cells and normal cells and to spatially resolve the different cell types based on both OCR and ECAR. This is, to the best of our knowledge, the first description of a device capable of spatiotemporally resolving OCR and ECAR at high resolution in cell cultures. The described method will be valuable for different in vitro applications in the investigation of cellular metabolism while also laying the foundation for the development of novel diagnostic tools for HNSCC patients.

## Supplementary Information

**Additional file 1. **pO_2_ measurements in cell colonies, **A, B** Individual Kyse30 (A) or FaDu (B) cells grown into different size colonies. On the left, brightfield micrographs are shown with the colony size indicated below. On the right, heatmaps represent pO_2_ after 10, 40 and 60 minutes (A) or 2, 30 and 60 minutes (B). Scale bars: 1 mm **C,D** Individual Kyse30 (C) or FaDu (D) cells expressing mCherry grown into different size colonies on a layer of HFF cells. On the left, micrographs with the brightfield and mCherry signal overlaid are shown. Kyse30/FaDu colonies are outlined in black or white and were overlaid with the heatmaps representing the pO_2_ after 30 and 50 minutes (C) or 2 and 10 minutes (D).

**Additional file 2. **pH measurements in cell colonies. **A, B** Individual Kyse30 (A) or FaDu (B) cells grown into different size colonies. On the left, brightfield micrographs are shown with the colony size indicated below. On the right, heatmaps represent pH after 10, 30 and 60 minutes. Scale bars: 1 mm **C,D** Individual Kyse30 (C) or FaDu (D) cells expressing mCherry grown into different size colonies on a layer of HFF cells. On the left, micrographs with the brightfield and mCherry signal overlaid are shown. Kyse30/FaDu colonies are outlined in black or white and were overlaid with the heatmaps representing the pH after 15 and 40 minutes (C) or 5 and 15 minutes (D).

## Data Availability

The datasets used and/or analysed during the current study are available from the corresponding author on reasonable request

## Abbreviations

2D: Two-dimensional
2-DG: 2-Deoxy-d-Glucose
BF: Bright field
ECAR: Extracellular acidification rate(s)
ESCC: Oesophageal squamous cell carcinoma(s)
FCCP: Carbonyl cyanide-4-(trifluoromethoxy)phenylhydrazone
Gluc: Glucose
HFF: Human foreskin fibroblast(s)
HNEpC: Human nasal epithelial cell(s)
HNSCC: Head and neck squamous cell carcinoma(s)
k: Kilo (thousand)
STO_2_p-Q: Spatiotemporal O_2_ and pH quantification
NHE1: Sodium-hydrogen antiporter 1
OCR: Oxygen consumption rate(s)
Oligo: Oligomycin
pH_e_: Extracellular pH
pH_i_: Intracellular pH
pO_2_: Partial oxygen pressure
PSM: Positive surgical margin(s)
ROI: Region(s) of interest
