# Supplementary figures and images for: High-resolution spatiotemporal pHe and pO2 imaging in head and neck and oesophageal carcinoma cells
Source: Cancer Metab. 2021 May 4;9:21. doi: 10.1186/s40170-021-00257-6 (PMC8097870; doi:10.1186/s40170-021-00257-6)

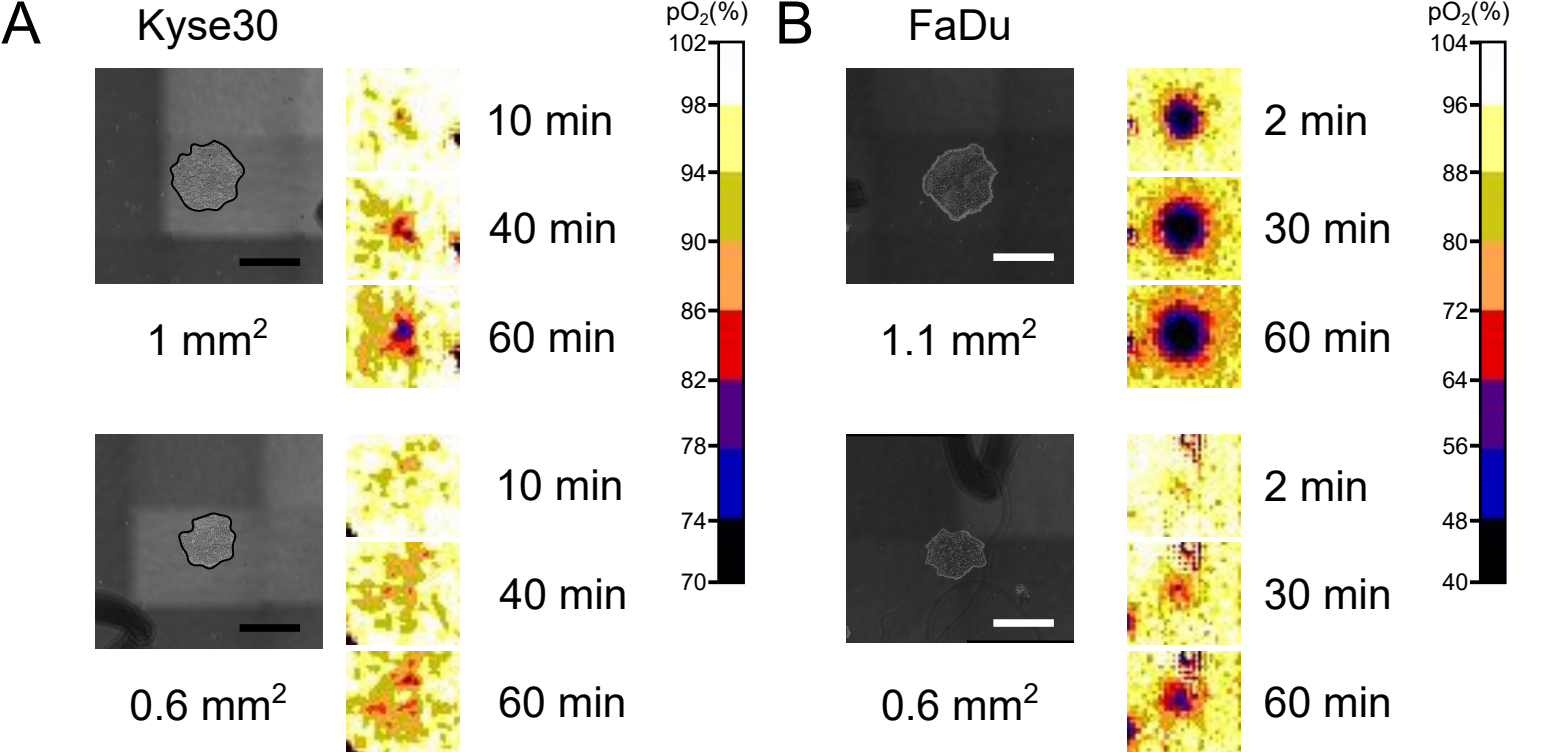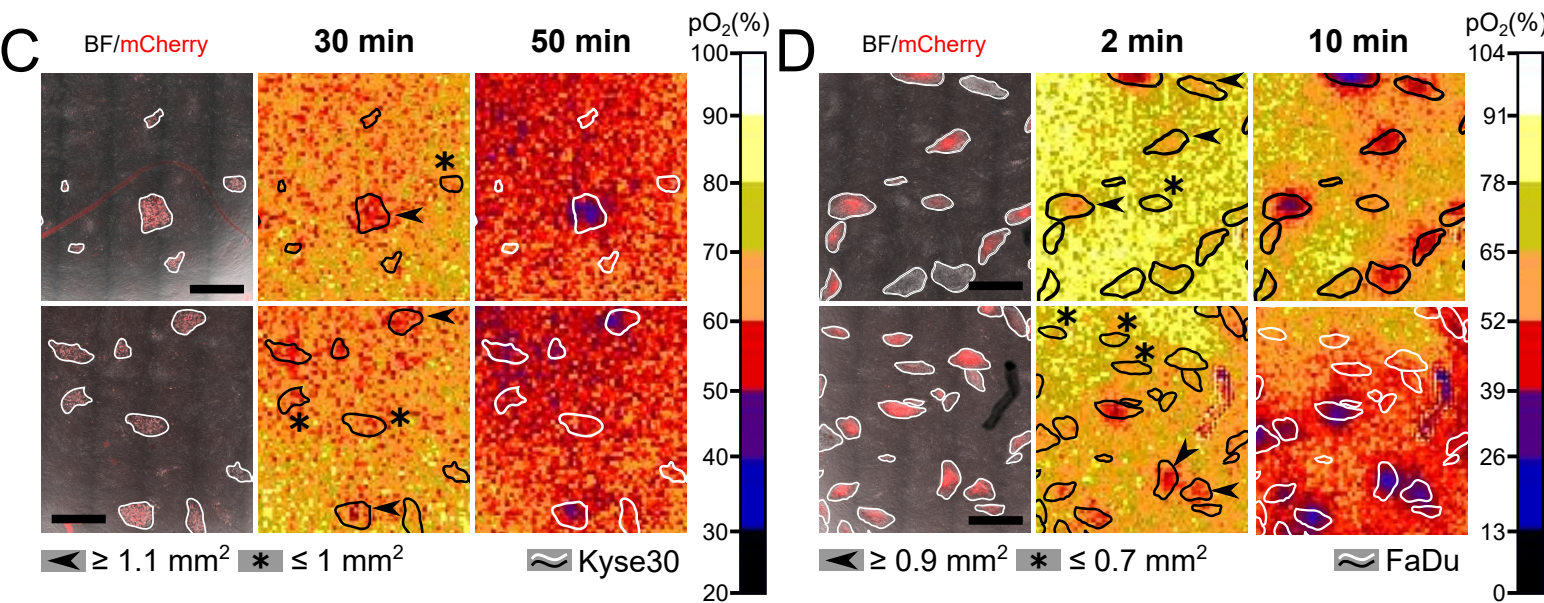

Supplement: Supplementary file 1 — Additional file 1. pO2 measurements in cell colonies, A, B Individual Kyse30 (A) or FaDu (B) cells grown into different size colonies. On the left, brightfield micrographs are shown with the colony size indicated below. On the right, heatmaps represent pO2 after 10, 40 and 60 minutes (A) or 2, 30 and 60 minutes (B). Scale bars: 1 mm C,D Individual Kyse30 (C) or FaDu (D) cells expressing mCherry grown into different size colonies on a layer of HFF cells. On the left, micrographs with the brightfield and mCherry signal overlaid are shown. Kyse30/FaDu colonies are outlined in black or white and were overlaid with the heatmaps representing the pO2 after 30 and 50 minutes (C) or 2 and 10 minutes (D). [file 40170_2021_257_MOESM1_ESM.pdf]

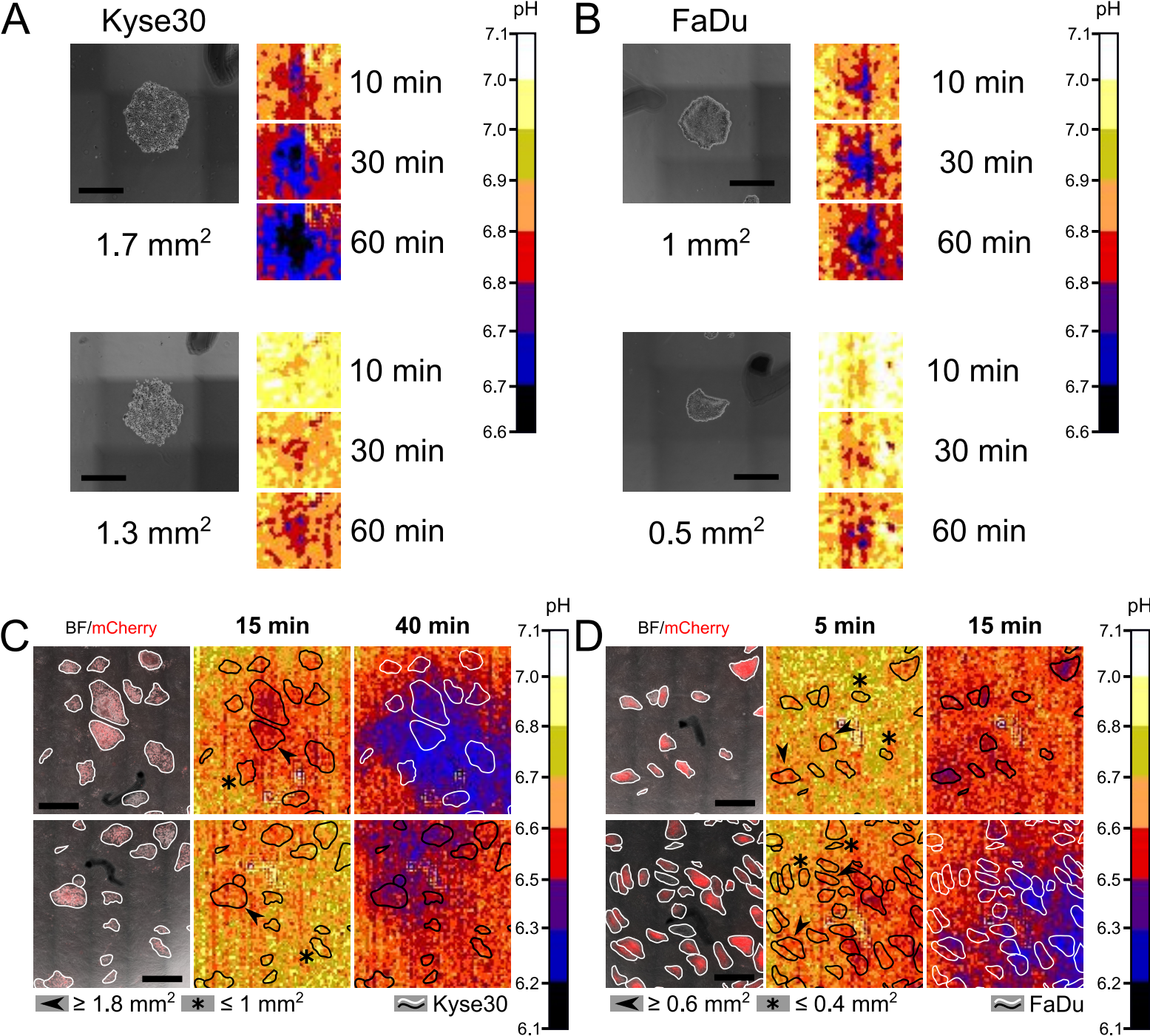

Supplement: Supplementary file 2 — Additional file 2. pH measurements in cell colonies. A, B Individual Kyse30 (A) or FaDu (B) cells grown into different size colonies. On the left, brightfield micrographs are shown with the colony size indicated below. On the right, heatmaps represent pH after 10, 30 and 60 minutes. Scale bars: 1 mm C,D Individual Kyse30 (C) or FaDu (D) cells expressing mCherry grown into different size colonies on a layer of HFF cells. On the left, micrographs with the brightfield and mCherry signal overlaid are shown. Kyse30/FaDu colonies are outlined in black or white and were overlaid with the heatmaps representing the pH after 15 and 40 minutes (C) or 5 and 15 minutes (D). [file 40170_2021_257_MOESM2_ESM.pdf]
